# Infection control management of patients with suspected highly infectious diseases in emergency departments: data from a survey in 41 facilities in 14 European countries

**DOI:** 10.1186/1471-2334-12-27

**Published:** 2012-01-28

**Authors:** Francesco M Fusco, Stefan Schilling, Giuseppina De Iaco, Hans-Reinhard Brodt, Philippe Brouqui, Helena C Maltezou, Barbara Bannister, René Gottschalk, Gail Thomson, Vincenzo Puro, Giuseppe Ippolito, EuroNHID Working Group

**Affiliations:** 1 National Institute for Infectious Diseases "L Spallanzani", Rome, Italy; 2 J. W. Goethe University, Frankfurt am Main, Germany; 3 IHU POLMIT CHU Nord AP-HM, Marseille, France; 4 Hellenic Center for Disease Control and Prevention, Athens, Greece; 5 Royal Free Hospital, London, UK; 6 Health Protection Authority, Frankfurt am Main, Germany; 7 Health Protection Agency, Porton Down, Salisbury, UK

## Abstract

**Background:**

In Emergency and Medical Admission Departments (EDs and MADs), prompt recognition and appropriate infection control management of patients with Highly Infectious Diseases (HIDs, e.g. Viral Hemorrhagic Fevers and SARS) are fundamental for avoiding nosocomial outbreaks.

**Methods:**

The EuroNHID (European Network for Highly Infectious Diseases) project collected data from 41 EDs and MADs in 14 European countries, located in the same facility as a national/regional referral centre for HIDs, using specifically developed checklists, during on-site visits from February to November 2009.

**Results:**

Isolation rooms were available in 34 facilities (82,9%): these rooms had anteroom in 19, dedicated entrance in 15, negative pressure in 17, and HEPA filtration of exhausting air in 12. Only 6 centres (14,6%) had isolation rooms with all characteristics. Personnel trained for the recognition of HIDs was available in 24 facilities; management protocols for HIDs were available in 35.

**Conclusions:**

Preparedness level for the safe and appropriate management of HIDs is partially adequate in the surveyed EDs and MADs.

## Background

Emergency Departments (EDs) and Medical Admission Departments (MADs) are high-risk areas for disease transmission in hospitals, since they are often overcrowded, and potentially infectious patients and susceptible individuals may wait in close proximity for several hours. Moreover, the identification and isolation of potentially infectious patients may be delayed, because of high work burden, lack of specific training and skills, or unavailability of adequate isolation procedures or areas [1]. In particular, Highly Infectious Diseases (HIDs, see definition in Additional file 1: Annex 1) pose a special risk for nosocomial outbreaks, if not adequately isolated and appropriately managed [2,3].

The European Network for Highly Infectious Diseases (EuroNHID) project, a 42-month (July 2007-December 2010) European Commission co-funded network, aims to enhance and maintain co-operation, and exchange of information and experiences on HIDs management among infectious disease clinicians, and to enhance preparedness and response to health threats from these diseases within Europe, whether naturally occurring, or deliberately released. EuroNHID includes 16 European Countries (Austria, Bulgaria, Denmark, Finland, France, Germany, Greece, Ireland, Italy, Luxembourg, Malta, Norway, Poland, Slovenia, Spain, and United Kingdom), is managed by a Coordination Team, based at the National Institute for Infectious Diseases "Lazzaro Spallanzani", Rome, Italy.

From February to November 2009, EuroNHID performed a survey in 48 isolation facilities identified by National Health Authorities as referral centres for the management of imported or autochthonous cases of HIDs [4]. Among these, 41 in 14 countries reported to have an ED or a MAD operating in the same hospital. The aim of this paper is to present data about logistic and infrastructures, infection control procedures, and availability of staff for the appropriate management of HIDs in these EDs and MADs. Moreover, indications for the adequate management of HIDs in these settings are given.

## Methods

A cross-sectional study has been performed, in order to investigate resources and capabilities for the management of HIDs in 41 EDs and MADs in 14 countries.

### Setting and participants

National health authorities in all European countries were contacted by the Coordination Team and by the European Commission, in order to suggest a physician with expertise in HID management as project partner. This process led to the inclusion of 15 countries, while a Norwegian isolation facility later joined the group after direct request from the Coordination Team. Most partners are clinicians working in isolation facilities designated for referral of patients with HIDs. Their areas of expertise include infectious diseases, intensive care, infection control, pulmonary medicine, occupational health, epidemiology and public health.

In order to survey only isolation facilities identified by national health authorities for the referral and management of HIDs, we asked partners to provide official documents in which these hospitals are clearly indicated. This process led to the identification of 48 facilities, which represent all identified centres for all participating countries except Spain, from which centres from Catalonia only were identified.

Forty-one isolation facilities in 14 countries reported to have an ED or a MAD operating in the same medical centre. We define an ED as the department of the hospital responsible for the provision of medical and surgical care to patients in need of immediate care arriving at the hospital. The MAD is the department, usually open round the clock, through which patients are admitted to the hospital. MADs can operate for planned admissions only, or for self-referring patients also, but usually are not able to provide immediate care. Among the 41 centres surveyed, some had an ED or a MAD only, and some had both departments: in these cases, we surveyed the department serving self-referring patients mainly, because our goal was to identify settings where patients with HIDs were most likely to be unrecognized.

### Data collection

Data were collected during on-site visits, using a set of checklists specifically developed. Three checklists were developed, including 16 main issues and 148 specific questions. Management of HIDs in EDs and MADs represents one of the main issues in checklist 1. The checklists are available on the website http://www.eunid.eu, after registration. All on-site visits were performed by the Project Coordinator together with a representative of the surveyed facility, during the period February-November 2009. All EDs and MADs were visited, except 2: in these cases, only fulfilled checklists were available.

### Outcomes and data analysis

In order to assess the status of each surveyed facility, a standard Evaluation Form was developed, on the basis of a literature review and the partners' expert opinion.

For the literature review data published up to June, 2011, were obtained by searches of PubMed and Medline, and from review of the references listed in retrieved articles, using as search term "Emergency Services, hospital" as MeSH, coupled with general terms such as "Civil Defense", "Bioterrorism" and "Hospital Preparedness" and with the name of each HID included as MeSH term. No data restrictions were placed on our searches.

In the Evaluation Form all data were summarized in 3 topics: availability and adequacy of isolation room(s), of infection control procedures, and of strategies for early recognition of HIDs. The level of adequacy for each topic is assessed by the EuroNHID expert panel: in particular about isolation room(s), the panel defined as adequate the availability of at least one isolation room equipped with at least one logistic or technical feature as listed in the Table 1; as partially adequate the availability of at least one isolation room without specific logistic/technical features; as inadequate the lack of an isolation room. About infection control procedures, the presence of all explored features and procedures as listed in the Table 2 was defined as adequate; the presence of at least 3 explored features and/or procedures as partially adequate; and 2 or less features and procedures as not adequate. Finally, early recognition strategies were defined as adequate if trained triage staff or other procedures are in place on a 24 hour-basis, partially adequate if these staff/procedures are not continuously in place, not adequate if not in place at all. The Evaluation Form is available at http://www.eunid.eu, after registration. Two members of the Coordination Team, including the Project Coordinator, applied this Evaluation Form for each surveyed facilities, in order to identify all critical points, and suggest affordable solutions. These Evaluation Forms were sent to the contact persons at the surveyed facilities, asking for feedback.

**Table 1:** Availability of isolation rooms and their logistic and technical features in 41 EDs and MADs 14 European Countries

	No. (%)
Number of EDs/MADs surveyed	41 (100)

Number of EDs/MADs with availability of isolation room(s)	34 (82,9)

*Logistic and technical features of these room(s)*	

Availability of a dedicated entrance directly from outside	15 (36,6)

Presence of anteroom	19 (46,3)

Availability of negative pressure	17 (41,5)

Dedicated ventilation system	16 (39,0)

HEPA filtration of exhausted air	12 (29,3)

Number of EDs/MADs with isolation room(s) equipped with all explored features	6 (14,6)

**Table 2:** Availability of infection control procedures in 41 EDs and MADs in 14 European Countries

	No. (%)
Number of EDs/MADs surveyed	41 (100)

*General infection control procedures*	

Availability of a general waiting area large enough for the safe distancing between attending persons (at least 1 meter/3 feet)	14 (34,1)

Availability of a reserved/separated waiting areas for suspected patients (e.g. patients with fever and cough)	22 (53,6)*

Availability of plans for the implementation of waiting areas if necessary	28 (68,3)

*Specific infection control procedures for suspected HIDs*	

Availability of (or easy access to) specific PPE	38 (92,7)

Availability of specific protocols for the management of suspected HIDs	35 (85,4)
	
including criteria for initial diagnostic suspect	32 (78,0)
	
including initial diagnostic work-up	27 (65,8)
	
including basic infection control measures	35 (85,4)
	
including initial medical treatment	20 (48,8)
	
including steps for alerting and notifying	32 (78,0)

Existence of a dedicated route from ED/MAD to isolation ward for suspected HIDs	25 (61,0)
	
if yes, by-passing other common areas	21 (51,2)
	
if yes, the transport is performed by:	
	
stretcher isolator	8 (19,5)
	
special ambulance through an external pathway	3 (7,3)
	
different procedures depending on risk assessment	9 (21,9)
	
with a normal stretcher, without special procedures	5 (12,2)

* not routinely used in all surveyed EDs/MADs

### Development of recommendations

On the basis of the selected literature, partners' expert opinion, and data collected during the surveys, EuroNHID developed indications for the adequate management of HIDs in EDs and MADs. These recommendations were discussed with all partners, and a consensus agreement was reached during the final meeting, in Rome in May 2010.

## Results

From February to November 2009, 41 EDs/MADs in 14 European Countries were surveyed. In the 41 centres included in our study, 26 had a general ED only, while 4 only had a MAD. In the remaining 11, where both departments were present, self-referring patients with suspected infectious diseases are referred to the MAD in 2 cases, while in the remaining 9 self-referring patients are referred to the general EDs. Consequently, our study includes 35 EDs and 6 MADs. Given the few MADs, data are not presented separately.

**Figure 1:**
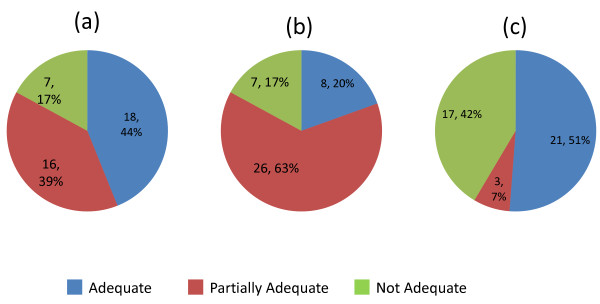
**Distribution of level of adequacy for (a) isolation rooms, (b) features and procedures for infection control, (c) strategies for early recognition of HIDs (specifically trained staff or other procedures), in 41 EDs and MADs**.

Thirty-four facilities had at least one dedicated room for the rapid isolation and evaluation of patients with suspected HIDs. In 15 facilities these were standard rooms. In the remaining facilities the rooms were equipped with at least one specific logistic feature or at least one technical feature. The availability of these features is reported in Table 1, and the level of adequacy in the explored EDs and MADs is showed in the Figure 1.

The availability of infection control procedures and equipments were addressed in the checklists, results are summarized in Table 2. The Figure 1 shows the level of adequacy of infection control in the surveyed EDs and MADs.

The project explored the availability of triage staff specifically trained for the early recognition of suspected HID patients, or alternatively the existence of other procedures for the early identification of these patients, such as a syndromic approach. Out of the 41 facilities, 21 have triage personnel with a specific training and background for the early identifying of suspected patients, or other procedures in place for this purpose. In 3 facilities, only some of triage staff have this expertise, thus the early recognition capability is not available on 24-h basis. Finally, the remaining 17 EDs and MADs have not specific strategies for the early recognition of suspected HID patients. The Figure 1 shows the level of adequacy of this topic in surveyed EDs and MADs.

## Discussion

### Brief history of preparedness in EDs and MADs

After September 11 and the "anthrax letter" attacks in the US, plans and strategies for the early recognition of HID patients (such as those with suspected VHFs or with smallpox-like symptoms), and for the management of infectious diseases outbreaks (e.g. due to bioterrorism attack) have been promoted in EDs and MADs, both in USA and in many European countries. All surveys conducted in the aftermath of these events demonstrated severe shortcomings in preparedness [5,6]. A study addressing the availability of preparedness plans before and after September 11, showed significant improvements [7]. The SARS outbreak in 2003 also dramatically changed the approach to isolation and infection control in EDs and MADs [8], and after its emerging the Centers for Disease Prevention and Control, and afterward the World Health Organization, issued new infection control guidelines, introducing the respiratory hygiene/cough etiquette measures as part of standard precautions [9,10]. Some years after these events, the Influenza A(H1N1) pandemic in 2009 became a test-bed, with encouraging results. Indeed, many EDs and MADs reported the adoption of interventions for the management of surge, including rapid systems for triage, logistic modifications of waiting and evaluation area, revised infection control procedures, and modification of staff number and roles [11-14]. It is, however, likely that there may be reporting bias of successful experiences. In real life, it is likely that the presence of plans did not assure their consistent application: a survey conducted in Atlanta, Georgia, after the 2009 pandemic, in 26 EDs revealed that, despite most (92%) of the facilities having pandemic influenza plans, 6 reported "overcrowding," 1 reported "severe overcrowding," and 2 pediatric EDs reported "dangerous overcrowding". Moreover, many reported various space limitations including an insufficient number of treatment rooms, insufficient waiting areas, or lack of space to designate a separate waiting room [15].

After these experiences, it is now clear that EDs and MADs represent a key setting for the management of infectious diseases emergencies. Indeed, they serve as the frontline for patients acutely entering the health care system, and their personnel are the guardians at the gate. It is widely recognized, also, that an important role of EDs and MADs during an infectious diseases emergencies would be to identify sentinel cases involved in the event, or the isolated case suspected to be affected by an HID; while other tasks include an important infection control role, the appropriate triage, the staff protection, the initial diagnostic and therapeutic approaches, and the coordination with external emergency response and public health authorities [16].

### Limits of our study

A limit of our study is represented by the fact that surveys mainly were performed before August 2009. This means that we collected most of data before that EDs and MADs experienced the surge of cases due to the Influenza A (H1N1) pandemic, that peaked in November 2009. The experience gained during the pandemic may have caused modifications and improvements of procedures and capabilities, not registered in our data.

As the main target of the project were isolation facilities for HID cases, the EDs and MADs surveyed are operating within, or in the same hospital compound, as a regional/national reference hospital for infectious diseases: thus, we believe that the occurrence of HIDs is more likely in these facilities, due to referral of suspected patients from other medical facilities or to self-referral of patient returning from endemic countries for HID. This may affect the external validity of our results as the surveyed facilities probably have put more emphasis on isolation and infection control issues than ordinary hospitals which may also have to handle cases with a HID.

Moreover, we only collected data about the availability of procedures, but we didn't assess their appropriateness or their application in the real-life. Similarly, we didn't collected data about the contents, and the completeness, of staff training.

The indications for adequate management have some limits, also. Given the infrequency of suspected and confirmed HIDs, no high-quality studies exist, or in some cases no studies at all. Consequently, no evidence-based recommendations, neither any system of ranking of recommendations, is possible. Therefore our indications are based on experiences reported in the literature or revealed during the surveys, and on the partners' expert opinion.

### Interpretation of our results

Despite these limits, some comments are possible. In most of surveyed EDs and MADs at least one isolation room for the isolation and evaluation of suspected HID patients is available, but their logistic and technical level is generally not adequate. Indeed, only 6 have rooms with all explored items, and rooms with a minimal technical requirement for isolation according to modern standards (negative pressure, anteroom and HEPA filtration of exhausting air) are present in 9 facilities only. In the remaining 32, isolation rooms are not present, or not fully adequate. Based on international guidelines [9,10,17-21], it is our opinion that the following features are essential for safe and effective isolation: negative pressure is necessary for the isolation of patients with confirmed or suspected diseases with obligate airborne transmission (such as XDR-TB), as well as for the effective isolation of patients with suspected or confirmed diseases with opportunistic airborne transmission, such as SARS, human-adapted highly pathogenic strains of influenza virus and smallpox; the presence of an anteroom increases the efficiency of the system, providing an obstacle against pressure loss, and provides a controlled environment in which donning and removal of PPE and other procedures can be done safely; finally the use of HEPA filtration for exhausting air is important in order to protect the environment and the persons around the room.

Infection control procedures are generally available in the surveyed EDs and MADs. The majority of surveyed EDs and MADs have logistically adequate waiting areas, or procedures for surge capacity. Indeed, in order to reduce the risk of spreading of infectious diseases, adequate distancing among waiting persons, or the use of a dedicated area for coughing and sneezing patients, is very important. We also explored the availability of procedures for the early management of patients suspected to be affected by HIDs: these procedures, which are not in place in 14,6% of surveyed EDs and MADs, are mainly focused on early recognition, isolation and infection control, and on steps for alerting and notifying the case. However, most of these procedures do not include strategies for initial diagnostic work-up and treatments, that are not considered by EDs and MADs their responsibility. However, we believe that certain diagnostic tests and treatments can be performed by the EDs and MADs. These include tests to rapidly exclude the most common causes of fever, such as malaria, in patients coming from endemic areas. Conveniently, the vast majority of EDs and MADs have easy access to specific PPE, such as FFP2 respirators.

All explored features and procedures are not enough for a safe management of these patients, if the staff is not sufficiently trained and skilled. Indeed, the effectiveness of protocols for the early recognition relies upon their correct application, or upon the staff awareness of potentially infected patients. According to our data, specifically trained triage staff are lacking in 41,5% of surveyed EDs and MADs, and in 7% these staff are not continuously available. Thus, despite that fact that these facilities are located in the same centre as a regional/national reference centre for infectious diseases, in about the half of them a patient with an HID could be unrecognized.

Overall, the preparedness status of the EDs and MADs surveyed is only partially adequate, and this is more surprising considering their location.

### Indications for adequate management

Different interventions should be promoted for an appropriate infection control management in EDs and MADs. Basically, standard precautions, including respiratory hygiene and cough etiquette measures, plus transmission-based precautions, should be implemented as completely as possible. The triage procedures should not only include an assessment of disease severity/urgency, but should consider, wherever possible, also the risk of disease transmission posed by the patient. A brief epidemiological investigation of patients with symptoms consistent with an HID may help in the rapid identification of suspected patients. Simple standardized forms should be available for rapid use by triage personnel, and should include (i) a brief travel history, (ii) an occupational history (e.g. the patient is an HCW, a veterinarian, a laboratory worker, a farmer), (iii) a contact history of exposure to other persons with similar illness; and (iv) the history of being part of a cluster. These patients should be placed in separate waiting/evaluation areas, if available, or removed as soon as possible from common areas. Once identified as a suspected patient, detailed procedures should be available and rigorously applied. These procedures should include at least the basic steps for the infection control measures to be applied (isolation, PPE to be used, disinfection issues if needed), and the actions for the activation and alerting of the response chain. These procedures could also include, if appropriate, the basic diagnostic work-up to be applied, and therapeutic interventions. The availability of technically well-equipped and logistically adequate isolation rooms is fundamental. These rooms should have a separate access directly from outside, or be logistically isolated from other common areas, and should be equipped with an anteroom. Ideally, these rooms should have negative pressure, HEPA filtration of exhausting air, sealing of windows and door, and surfaces inside should be easy to decontaminate. Finally, all HCWs, or at least dedicated personnel depending on EDs policies, should be familiar with PPE use, donning and removal, isolation procedures and disinfection issues, as well as with the alert and command chain. Recommendations about core-curriculum for HCWs dealing with HIDs have been proposed [22], and should be adapted by responsible persons to ED and MAD settings. Triage HCWs, in particular, should be specifically trained in the recognition of suspected patients. This also implies a continuous updating on outbreaks ongoing in the world. Operatively, one HCW (from ED/MAD or from Infectious Diseases department according to local policies) should be responsible to monitor the major on-line epidemiological alerts sites and bulletins, and to disseminate the news to the triage staff.

The application of optimal requirements summarized in the Additional file 1: Annex 2, including the availability of a technically and logistically adequate isolation room, should be reserved to those EDs and MADs where it is more likely to have patients with HIDs (such as the EDs with an high average number of patients located in the capitals or nearby international airports/ports, those located in specialist Infectious Diseases hospitals or in the same centre as an isolation facilities, those located in endemic areas for diseases of interest), because of the cost of some of these interventions. On the opposite, the minimal requirements suggested in the Additional file 1: Annex 2 should be applied in all EDs/MADs.

## Conclusions

Despite the fact that health threats due to HIDs are constantly present, our survey reveals that the general preparedness level in surveyed EDs and MADs is only partially adequate. This is a cause for concern as the surveyed facilities are located in the same hospital as the respective regional/national reference centers for HIDs. Interventions to improve the capacity for early recognition and appropriate management if HIDs in EDs and MADs are strongly advised.

## Competing interests

The authors declare that they have no competing interests.

## Authors' contributions

FMF drafted the manuscript, substantial contributed to design the study, participated in the acquisition and analysis of data, and gave the final approval of the version to be published; SS, GDI, HRB, PB, HCM, BB, RG, GT and VP substantial contributed to design the study, participated in the acquisition and interpretation of data, and gave the final approval of the version to be published; GI substantial contributed to design the study, and gave the final approval of the version to be published. All authors read and approved the final manuscript.

## Pre-publication history

The pre-publication history for this paper can be accessed here:

http://www.biomedcentral.com/1471-2334/12/27/prepub

## Supplementary Material

Additional file 1 **Annex 1**. Definition and list of Highly Infectious Diseases (HIDs). **Annex 2 **Indications for the safe and appropriate management of suspected HID patients in EDs and MADs. Click here for file

## References

[B1] Rothman RE Irvin CB Moran GJ Sauer L Bradshaw YS Fry RB Jr Josephson EB Ledyard HK Hirshon JM Public Health Committee of the American College of Emergency Physicians Respiratory hygiene in the emergency department Ann Emerg Med 2006 48 5 570 582 10.1016/j.annemergmed.2006.05.018 17052558 PMC7115302

[B2] Bannister B Puro V Fusco FM Heptonstall J Ippolito G EUNID Working Group Framework for the design and operation of high-level isolation units: consensus of the European Network of Infectious Diseases Lancet Infect Dis 2009 9 1 45 56 10.1016/S1473-3099(08)70304-9 19095195 PMC7185791

[B3] Brouqui P Puro V Fusco FM Bannister B Schilling S Follin P Gottschalk R Hemmer R Maltezou HC Ott K Peleman R Perronne C Sheehan G Siikamäki H Skinhoj P Ippolito G EUNID Working Group Infection control in the management of highly pathogenic infectious diseases: consensus of the European Network of Infectious Disease Lancet Infect Dis 2009 9 5 301 311 10.1016/S1473-3099(09)70070-2 19393960 PMC7106353

[B4] Fusco FM Schilling S Puro V Brodt HR Follin P Jarhall B Bannister B Maltezou HC Thomson G Brouqui P Ippolito G EuroNHID Study Group EuroNHID checklists for the assessment of high-level isolation units and referral centres for highly infectious diseases: results from the pilot phase of a European survey Clin Microbiol Infect 2009 15 8 711 719 10.1111/j.1469-0691.2009.02874.x 19486074

[B5] Wetter DC Daniell WE Treser CD Hospital preparedness for victims of chemical or biological terrorism Am J Public Health 2001 91 5 710 716 11344876 10.2105/ajph.91.5.710 PMC1446687

[B6] Schultz CH Mothershead JL Field M Bioterrorism preparedness. I: The emergency department and hospital Emerg Med Clin North Am 2002 20 2 437 455 10.1016/S0733-8627(02)00003-2 12120486

[B7] Braun BI Darcy L Divi C Robertson J Fishbeck J Hospital bioterrorism preparedness linkages with the community: improvements over time Am J Infect Control 2004 32 6 317 326 15454887 10.1016/j.ajic.2004.01.003

[B8] Drosten C Chun-Wing Lau A Preiser W Kit-Ying So L Yin-Chun Yam L SARS Reference - 10/2003 http://www.SARSReference.com

[B9] Centers for Disease Control and Prevention Guideline for isolation precautions: preventing transmission of infectious agents in healthcare settings http://www.cdc.gov/hicpac/pdf/isolation/isolation2007.pdf

[B10] World Health Organization Infection prevention and control of epidemic- and pandemic-prone acute respiratory diseases in health care, 2007 http://www.who.int/csr/resources/publications/WHO_CDS_EPR_2007_6c.pdf 24983124

[B11] Cooper MC Walz K Brown MG McDonald K Dwyer D Dowd P Byrne K Griffin M Boston medical center pediatric emergency response to H1N1 J Emerg Nurs 2009 35 6 580 583 10.1016/j.jen.2009.07.011 19914494

[B12] Bellazzini MA Minor KD ED syndromic surveillance for novel H1N1 spring 2009 Am J Emerg Med 2011 29 1 70 74 10.1016/j.ajem.2009.09.009 20825786 PMC3042301

[B13] Boehm N Cabral M Hankinson M Sakers C H1N1 2009: one pediatric emergency department's experience J Emerg Nurs 2010 36 2 125 129 10.1016/j.jen.2009.07.016 20211403

[B14] Rodriguez-Noriega E Gonzalez-Diaz E Morfin-Otero R Gomez-Abundis GF Briseño-Ramirez J Perez-Gomez HR Lopez-Gatell H Alpuche-Aranda CM Ramírez E López I Iguala M Bojórquez Chapela I Palacios Zavala E Hernández M Stuart TL Villarino ME Widdowson MA Waterman S Uyeki T Azziz-Baumgartner E de Guadalajara Hospital Civil, Fray Antonio Alcalde Emerging Respiratory Infections Response Team Hospital triage system for adult patients using an influenza-like illness scoring system during the 2009 pandemic--Mexico PLoS One 2010 5 5 e10658 10.1371/journal.pone.0010658 20498718 PMC2871038

[B15] Sugerman D Nadeau KH Lafond K Cameron W Soetebier K Jhung M Isakov A Greenwald I Neil K Schrag S Fry A A survey of emergency department 2009 pandemic influenza A (H1N1) surge preparedness-Atlanta, Georgia, July-October 2009 Clin Infect Dis 2011 52 Suppl 1 S177 S182 21342892 10.1093/cid/ciq035 PMC5772599

[B16] Macintyre AG Christopher GW Eitzen E Jr Gum R Weir S DeAtley C Tonat K Barbera JA Weapons of mass destruction events with contaminated casualties: effective planning for health care facilities JAMA 2000 283 2 242 249 10.1001/jama.283.2.242 10634341

[B17] Smith PW Anderson AO Christopher GW Cieslak TJ Devreede GJ Fosdick GA Greiner CB Hauser JM Hinrichs SH Huebner KD Iwen PC Jourdan DR Kortepeter MG Landon VP Lenaghan PA Leopold RE Marklund LA Martin JW Medcalf SJ Mussack RJ Neal RH Ribner BS Richmond JY Rogge C Daly LA Roselle GA Rupp ME Sambol AR Schaefer JE Sibley J Streifel AJ Essen SG Warfield KL Designing a biocontainment unit to care for patients with serious communicable diseases: a consensus statement Biosecur Bioterror 2006 4 4 351 365 10.1089/bsp.2006.4.351 17238819

[B18] Jensen PA Lambert LA Iademarco MF Ridzon R CDC. Guidelines for preventing the transmission of Mycobacterium tuberculosis in health-care settings, 2005 MMWR Recomm Rep 2005 54 1 141 16382216

[B19] Sehulster L Chinn RY CDC, HICPAC Guidelines for environmental infection control in health-care facilities. Recommendations of CDC and the Healthcare Infection Control Practices Advisory Committee (HICPAC) MMWR Recomm Rep 2003 52 1 42 12836624

[B20] Ninomura P Rousseau C Bartley J Updated guidelines for design and construction of hospital and health care facilities, 2006 http://www.newcomb-boyd.com/pdf/ASHRAE%20rousseau%20et%20al.pdf

[B21] Booth TF Kournikakis B Bastien N Ho J Kobasa D Stadnyk L Li Y Spence M Paton S Henry B Mederski B White D Low DE McGeer A Simor A Vearncombe M Downey J Jamieson FB Tang P Plummer F Detection of airborne severe acute respiratory syndrome (SARS) coronavirus and environmental contamination in SARS outbreak units J Infect Dis 2005 191 9 1472 1477 10.1086/429634 15809906 PMC7202477

[B22] Baka A Fusco FM Puro V Vetter N Skinhoj P Ott K Siikamaki H Brodt HR Gottschalk R Follin P Bannister B De Carli G Nisii C Heptonstall J Ippolito G European Network of Infectious Diseases A curriculum for training healthcare workers in the management of highly infectious diseases Euro Surveill 2007 12 6 E5 E6 17991402 10.2807/esm.12.06.00716-en

