# Supplementary material for: Infection control management of patients with suspected highly infectious diseases in emergency departments: data from a survey in 41 facilities in 14 European countries
Source: BMC Infect Dis. 2012 Jan 28;12:27. doi: 10.1186/1471-2334-12-27 (PMC3292988; doi:10.1186/1471-2334-12-27)
Supplement: Additional file 1 — Annex 1. Definition and list of Highly Infectious Diseases (HIDs). Annex 2 Indications for the safe and appropriate management of suspected HID patients in EDs and MADs. [file 1471-2334-12-27-S1.DOC]

| **Annex 1 – Definition and list of Highly Infectious Diseases (HIDs) [2,3]** |
| --- |
| Highly Infectious Diseases (HIDs) are those that are:   - being easily transmissible from person-to-person, - causing life-threatening illness, - presenting a serious hazard in health-care settings and in the community, requiring specific control measures.   The following agents/diseases are included among HIDs:   - Viral hemorrhagic fevers (VHFs, Marburgvirus, Ebolavirus, Crimean Congo hemorrhagic fever virus, Lassa virus, the recently-recognized Lujo virus, and South American haemorrhagic fever viruses - Junin, Machupo, Sabia, and Guanarito). - SARS Co-V - Multi drug- and extensively drug- resistant M. tuberculosis (MDR- and XDR-TB, known or suspected infection) - Newly emerging highly pathogenic strains of Influenzavirus - Smallpox and other Orthopox infections (eg Monkeypox, but excluding Vaccinia virus) - Other emerging highly pathogenic agents, including agents of deliberate release (eg pneumonic plague), some of which could also be extensively antibiotic-resistant. |

| **Annex 2 – Indications for the safe and appropriate management of suspected HID patients in EDs and MADs** |
| --- |
| *Optimal requirements*   - Implement as far as possible the application of routine and transmission-based precautions. In particular about respiratory hygiene/cough etiquette measures:   - Waiting areas should be large enough to allow people to sit at least one meter apart or dedicated areas for people with suggestive symptoms for transmissible diseases should be present;   - Visual signals (e.g. posters) should be present and well visible in common waiting areas;   - Disposable tissues and/or surgical masks should be available and should be offered to coughing and sneezing persons (including patients and visitors);   - Places for the disposal of used tissues should be present;   - Stations for hand hygiene, with all items needed, including distributors for alcohol-based solution, should be present in the EDs/MADs, especially in waiting rooms;   - If possible, an HCW should have responsibility for ensuring compliance with these measures; - Develop triage procedures including an assessment of transmission risk. This procedures should include a brief epidemiological investigation, that should be performed using standardized forms; - An isolation room should be available for the rapid isolation and initial assessment of patients with suspected HIDs. This room should be logistically and technically adequate; - Staff should be adequately trained, using existing recommendations for the HCWs dealing with HIDs, adapted for the specific settings of EDs and MADs. Ideally, all ED/MAD staff should be familiar with isolation and infection control procedures, and with correct management of Personal Protective Equipment; - HCWs dedicated to triage should be specifically trained in the rapid recognition of suspected patients, and a simple system for the updating of epidemiological information should be in place.   *Minimal requirements*   - Respiratory hygiene/cough etiquette measures should be applied in all EDs and MADs at all times, as described in the optimal requirements. If it is not possible to dedicate an HCWs to ensuring compliance, visual signs with instructions should be used; - If the use of dedicated waiting room is not possible, ask people with fever and cough/sneeze to sit at least 1 meter away from other patients, and develop plan for surge capacity in triage/waiting areas in case of over-crowding; - Include a basic epidemiological evaluation (exploring the travel and exposure history of the patient) into triage procedures, in order to identify patients more likely to be affected by HIDs; - Identify a room that could be used for isolation. Prefer those rooms that are peripherally located and near to alternative entrances, if available, in order to functionally isolate the area; - A core number of HCWs should be trained in the correct management of suspected HIDs’ patients. Shift plans should be developed in order to have some trained HCWs available in every moment, or procedures for their rapid alerting should be in place. Triage personnel should have basic training in the rapid recognition of suspected HIDs, and should have access to an epidemiological update about the main outbreaks ongoing in the world. |
